# Regulation of Serum Sphingolipids in Andean Children Born and Living at High Altitude (3775 m)

**DOI:** 10.3390/ijms20112835

**Published:** 2019-06-11

**Authors:** Pietro Barbacini, Josefina Casas, Enrica Torretta, Daniele Capitanio, Gustavo Maccallini, Valeria Hirschler, Cecilia Gelfi

**Affiliations:** 1 PhD school in Molecular and Translational Medicine, Università degli Studi di Milano, 20142 Milan, Italy; pietro.barbacini@unimi.it; 2 Department of Biomedical Sciences for Health, University of Milan, Luigi Mangiagalli 31, 20133 Milan, Italy; enrica.torretta@unimi.it (E.T.); daniele.capitanio@unimi.it (D.C.); 3 Research Unit on BioActive Molecules, Catalan Institute of Advanced Chemistry (IQAC/CSIC), Department of Biological Chemistry, CIBEREHD, Jordi Girona 18, E-08034 Barcelona, Spain; fina.casas@iqac.csic.es; 4 I.R.C.C.S Orthopedic Institute Galeazzi, R. Galeazzi 4, 20161 Milan, Italy; 5 Laboratorio Hidalgo, B1640EYA Buenos Aires, Argentina; gustavo.maccallini@laboratoriohidalgo.com; 6 Nutrition and Diabetes Division, University of Buenos Aires (UBA), 1006 Buenos Aires, Argentina; vhirschler@intramed.net

**Keywords:** high-altitude hypoxia, dyslipidemia, sphingolipids, ceramides, sphingosine-1-phosphate, sphingomyelins, LC-MS/MS

## Abstract

Recent studies on Andean children indicate a prevalence of dyslipidemia and hypertension compared to dwellers at lower altitudes, suggesting that despite similar food intake and daily activities, they undergo different metabolic adaptations. In the present study, the sphingolipid pattern was investigated in serum of 7 underweight (UW), 30 normal weight (NW), 13 overweight (OW), and 9 obese (O) Andean children by liquid chromatography-mass spectrometry (LC-MS). Results indicate that levels of Ceramides (Cers) and sphingomyelins (SMs) correlate positively with biochemical parameters (except for Cers and Vitamin D, which correlate negatively), whereas sphingosine-1-phosphate (S1P) correlates negatively. Correlation results and LC-MS data identify the axis high density lipoprotein-cholesterol (HDL-C), Cers, and S1P as related to hypoxia adaptation. Specifically UW children are characterized by increased levels of S1P compared to O and lower levels of Cers compared to NW children. Furthermore, O children show lower levels of S1P and similar levels of Cers and SMs as NW. In conclusion, our results indicate that S1P is the primary target of hypoxia adaptation in Andean children, and its levels are associated with hypoxia tolerance. Furthermore, S1P can act as marker of increased risk of metabolic syndrome and cardiac dysfunction in young Andeans living at altitude.

## 1. Introduction

Several studies have addressed the comprehension of molecular mechanisms at the basis of a high-altitude hypoxia adaptation of Andeans and Tibetans, allowing them to permanently inhabit at 3500–4500 m of altitude [1,2,3]. These two ethnic groups are characterized by different traits associated with adaptive responses to hypobaric hypoxia, however the molecular mechanisms are still not completely clear. Advanced genome-wide scanning identified genes and genetic variants that contribute to human adaptation to altitude in Tibetans [4,5] and Andeans [6,7], confirming different adaptive mechanisms. Specifically, Andeans appear to develop metabolic syndrome [8], which is not described in Tibetans, and they have elevated hemoglobin concentrations compared to Tibetans, suggesting a different trait in oxygen delivery and metabolism [9,10,11,12]. Hypoxia is a key factor involving most of the major biological pathways that contribute to respiratory dysfunctions, cardiovascular disorders and diabetes, and genetic studies of high altitude dwellers have focused on key genes of the hypoxia inducible factor (HIF) pathway that detect and react to oxygen shortage. Mutations and polymorphisms were identified in Endothelial PAS Domain Protein 1 (EPAS1) and Egl-9 Family Hypoxia Inducible Factor 1 (EGLN1), providing a gene signature of these populations [13,14]. Furthermore, a positive selection for nitric oxide synthase 2 (NOS2A) has been described [6] in Andeans, even though this pattern cannot fully explain discrepancies in body weight composition and dyslipidemia observed in this population [15,16].

Recent studies on Koya children (a community born and living in the Andean region of Salta Province, 3750 m a.s.l.) indicate a higher prevalence of dyslipidemia and hypertension compared to a similar background community living at lower altitudes in Chicoana (CH), 1400 m a.s.l. [16,17], suggesting that despite similar food intake and daily activities, Koya children undergo a different metabolic adaptation. Furthermore, a study performed in San Antonio de los Cobres (SAC; a city located at 3775 m a.s.l.) on school children showed a higher prevalence of dyslipidemia compared to urban Buenos Aires children (BA) living at sea level [8]. It must be pointed out that SAC children had higher triglyceride and lower HDL-C concentrations than BA children, with both groups belonging to a similar ethnicity, suggesting that exposure to altitude might be the main factor associated with dyslipidemia. Moreover, high atherogenic risk is also indicated by different lipid-related indexes, such as total cholesterol/HDL-C, triglycerides/HDL-C, and low density lipoprotein-cholesterol (LDL-C) LDL-C/HDL-C. Furthermore, higher levels of triglycerides/HDL-C index were associated with an increased proportion of small and dense LDL-C [18,19,20].

The aim of the present study is to unravel the contribution of sphingolipids in metabolic adaptation among Koya children born and living at altitude (3775 m a.s.l.), in order to have better insights into the metabolic characteristics of this group with different body weight composition, and to identify markers that are able to predict the development of cardiovascular disease and diabetes in adulthood.

It is known that the total cellular ceramide (Cers) concentration increases in cell models in response to various stresses, such as hypoxia and restricted blood supply (ischemia), and that sphingosine 1 phosphate (S1P) is an important signaling molecule derived from ceramide degradation [21,22].

Cers are synthesized through three different pathways: the *de novo* biosynthesis pathway from palmitoyl-CoA and serine; the sphingomyelin hydrolysis pathway (SMase pathway), which generates Cers from SMs; and the salvage pathway, which generates ceramides from the catabolism of complex glycosphingolipids. Biochemically, individual ceramide synthases (CerS) isoforms (from 1 to 6) show substrate preference for specific chain length fatty acyl-CoAs, thus generating ceramides with different acyl-chains, which have been associated with obesity and glucose intolerance [23]. Ceramide is converted to sphingosine by ceramidase, and then sphingosine is phosphorylated into S1P by sphingosine kinase (SphK) 1 or 2, located in the cytosol and in the nucleus, respectively [24,25,26]. S1P is quickly degraded by specific phosphatases and lyases localized in the endoplasmic reticulum (ER) [27].

The relationship between ceramides and a high-fat diet was demonstrated in mice, in which alterations of muscle ceramide levels and glucose tolerance were associated with a high-fat diet [28]. These alterations were ameliorated by treatment with myriocin, an inhibitor of *de novo* biosynthesis, highlighting the central role of ceramide in metabolic changes. Ceramide increment alters mitochondria membrane permeability, inhibits electron transport chain intermediates, and promotes mitochondria oxidative stress [29]. Furthermore, ceramides levels can be cause [30] and effector [31], leading to peripheral and central insulin resistance that contribute to diabetes [32].

To characterize the contribution of sphingolipids associated with hypoxia in obesity, we investigate their differential abundance of sphingolipids in serum of UW, NW, OW, and O Koya children by combining a single phase extraction method with high resolution LC-MS analyses and multiple reaction monitoring (MRM).

## 2. Results

### 2.1. Biochemical Parameters Assessment

Biochemical assessment is summarized in Table 1. UW and OW groups were characterized by mean levels of TC defined as acceptable by the National Cholesterol Educational Program [33], while NW and O subjects showed borderline levels for total cholesterol (TC). Acceptable levels of mean HDL-C were detected only in NW subjects, while other groups showed lower mean levels of HDL-C, classifying them as borderline [33]. LDL-C mean levels were acceptable for all groups, being lower than 110 mg/dL, whereas all groups showed borderline levels of mean triglycerides (TG), except O subjects, which showed high levels [34]. Mean levels of Vitamin D were assessed and all groups were deficient [35,36]. Mean glycaemia was beneath alert limits for all groups [34].

### 2.2. Anthropometrics and Biochemical Differences

Data from comparison of biochemical parameters and anthropometric characteristics are summarized in Table 1.

HDL-C was higher in NW subjects compared to O (*p*-value < 0.01), whereas TC/HDL-C ratio was higher in O subjects compared to UW and NW (*p*-value < 0.05 and 0.01, respectively). Homeostatic Model assessment for insulin resistance (HOMA-IR) and insulin were higher in obese and overweight subjects compared to NW (*p*-value < 0.01 and 0.05, respectively, in both comparisons).

### 2.3. Sphingolipids and Biochemical Parameters Correlation

**Figure 1:**
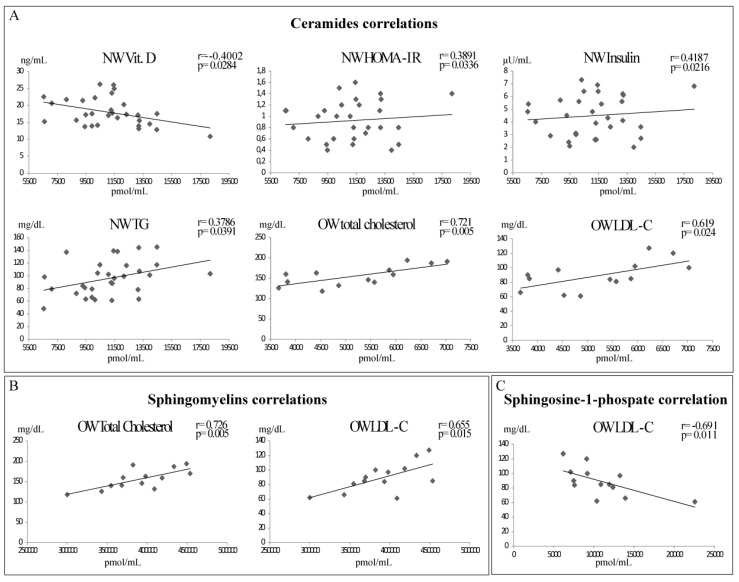
Total Ceramide (**A**), total sphingomyelin (**B**), and total S1P (**C**) scatter plots. Data for cers, SMs, and S1P are expressed as pmol/mL in log scale; data for Vitamin D are in ng/mL and data for insulin are in μU/mL; TG, TC, and LDL-C are in mg/dL; *p*-values from two tailed test, *r* value from Pearson’s correlation.

Scatter plots of total Cers, total SMs, and S1P levels with main biochemical parameters are summarized in Figure 1; a correlation table can be found in Supplementary Table S1.

UW subjects showed a positive correlation between SMs levels and HDL-C (*r* = 0.854, *p*-value = 0.015).

In NW subjects, Cers showed a positive correlation with HOMA-IR (*r* = 0.389, *p*-value= 0.0336), insulin (*r* = 0.418, *p*-value = 0.021), and TG (*r* = 0.379, *p*-value= 0.039), and a negative correlation with Vitamin D (*r* = −0.4, *p*-value = 0.028). In the same group of subjects, a negative correlation (*r* = −0.375, *p*-value = 0.041) was also identified between S1P and HDL-C, while no correlations were found for SMs.

In OW subjects, total Cers show a positive correlation with TC (*r* = 0.721, *p*-value = 0.005), LDL-C (*r* = 0.691, *p*-value = 0.024), as well as total SMs that strongly correlate with TC (*r* = 0.726, *p*-value = 0.005), LDL-C (*r* = 0.655, *p*-value = 0.015), and HDL-C (*r* = 0.603, *p*-value = 0.029), whereas S1P was negatively correlated with LDL-C levels (*r* = −0.691, *p*-value = 0.011).

In O subjects, a correlation was found comparing Cers and Glycaemia (*r* = 0.683, *p*-value = 0.043).

### 2.4. Sphingolipid Results by LC-MS

**Figure 2:**
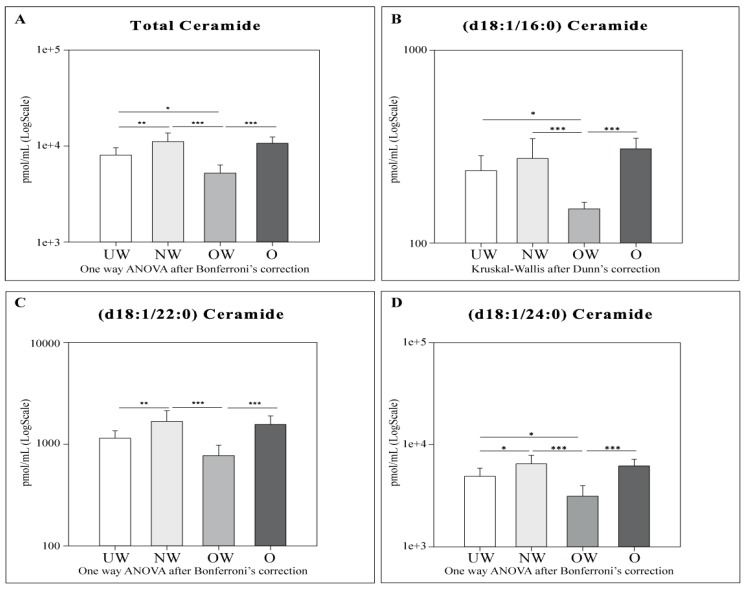
LC-MS results for total ceramides (**A**), Cer (d18:1/16:0) (**B**), Cer (d18:1/22:0) (**C**), and Cer (d18:1/24:0) (**D**). Data are expressed as pmol/mL in Log scale; *p* values are expressed as * *p*-value < 0.05, ** *p*-value < 0.01, and *** *p*-value < 0.001.

**Figure 3:**
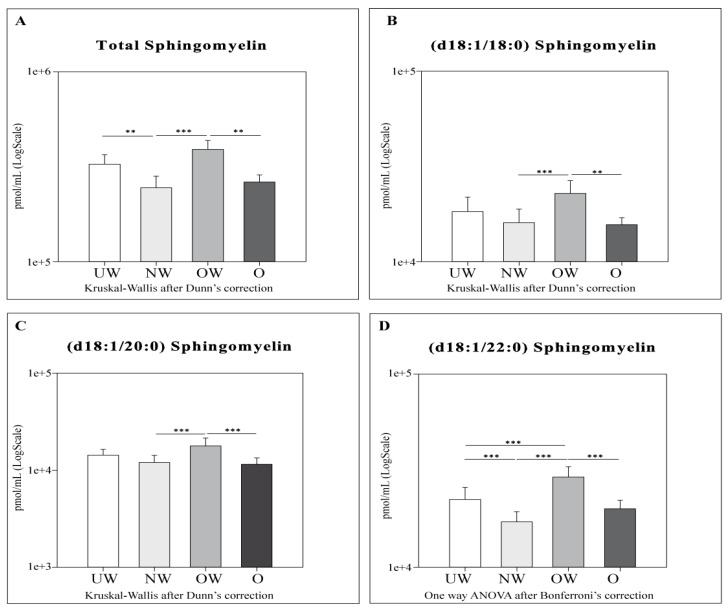
LC-MS results for total sphingomyelins (**A**), SMs (d18:1/18:0) (**B**), Cer (d18:1/20:0) (**C**), and Cer (d18:1/22:0) (**D**). Data are expressed as pmol/mL in Log scale; *p* values are expressed as ** *p*-value < 0.01, and *** *p*-value < 0.001.

**Figure 4:**
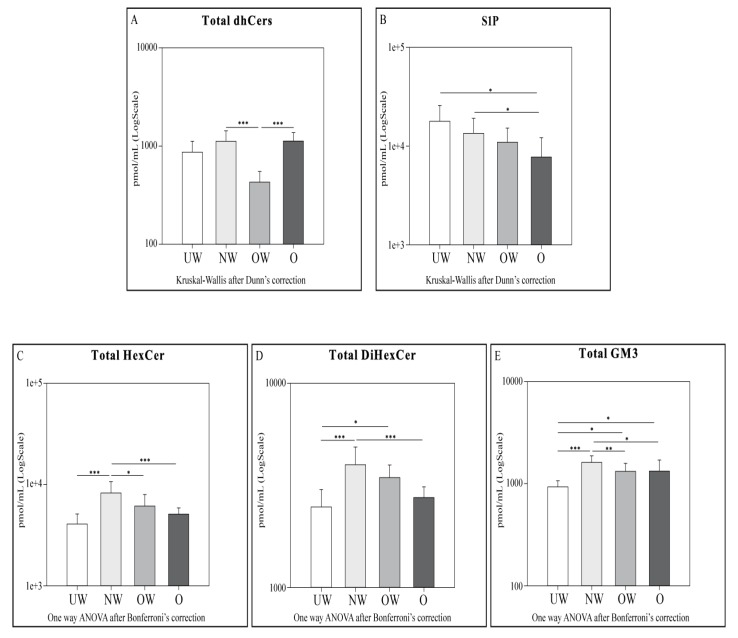
LC-MS and LC-MS/MS results for total dhCers (**A**), S1P (**B**), total HexCer (**C**), total dihexCer (**D**), and total GM3 (**E**). Data are expressed as pmol/mL in Log scale; *p* values are expressed as * *p*-value < 0.05, ** *p*-value < 0.01, and *** *p*-value < 0.001.

Cers (Figure 2), SMs (Figure 3), total dihydroceramides (dhCers), total hexosylceramides (HexCer), total dihexosylceramides (diHexCer), total monosialodihexosylganglioside (GM3), and total S1P levels (Figure 4) were assessed between different body mass index (BMI) percentile groups. In order to obtain a more refined characterization, Cers d18:1/16:0, d18:1/22:0, and d18:1/24:0 (Figure 2), and SMs d18:1/18:0, d18:1/20:0, and d18:1/22:0 (Figure 3) acyl chains were also investigated.

Total Ceramides were higher in NW subjects compared to UW and OW (*p*-value, respectively, < 0.01 and 0.001) subjects, and in O and UW compared to OW (*p*-value < 0.001 and 0.05, respectively). As expected, Cers acyl chains (d18:1/16:0, d18:1/22:0, and d18:1/24:0) followed the same trend as total Cers.

In particular, Cer (d18:1/16:0) was higher in UW, NW, and O subjects compared to OW (*p*-value < 0.05 and *p*-value < 0.001, respectively). Cer (d18:1/22:0) showed the same pattern as Cer (d18:1/16:0) in NW (*p*-value < 0.001) and O (*p*-value < 0.001) subjects compared to OW subjects. NW subjects also showed higher levels of Cer (d18:1/22:0) compared to UW (*p*-value < 0.01). Ceramide (d18:1/24:0) followed the same trend as total Cers, with higher levels in NW subjects compared to UW and OW (*p*-value < 0.05 and 0.001, respectively). Also, O and UW subjects showed higher levels of Cer (d18:1/24:0) when compared to OW subjects (*p*-value < 0.001 and 0.05, respectively).

Conversely, total SMs (Figure 3) were higher in OW subjects compared to O (*p*-value < 0.01) and NW (*p*-value < 0.001) subjects, and were also higher in UW compared to NW (*p*-value < 0.01).

SMs acyl chains followed the same trend highlighted for total SMs. In particular, SM (d18:1/18:0) was higher in OW subjects compared to both NW (*p*-value < 0.001) and O (*p* < 0.01); this increment was not observed in UW. SM (d18:1/20:0) was also higher in OW, comparing NW (*p*-value < 0.001) and O (*p*-value < 0.001) groups. SM (18:1/22:0) showed the same behavior of SM (d18:1/20:0) but significant differences were observed also in UW vs. NW, where higher levels were seen in the UW group (*p*-value < 0.001) and in UW vs. OW, where UW subjects were characterized by lower levels of SM (*p*-value < 0.001).

Regarding total dhCers (Figure 4), the same trend of Cers was identified—NW and O subjects showed higher levels of serum dhCers compared to the OW group (*p*-value < 0.001 in both comparisons).

LC-MS analysis for total glycosphingolipids HexCer, diHexCer, and GM3 (Figure 4) identified a characteristic pattern in all groups—NW patients have higher levels of the above-mentioned SLs species compared to UW (*p*-value < 0.001). For total HexCer, NW subjects showed higher levels also compared to OW and O (*p*-value < 0.05 and 0.001, respectively). For total diHexCer, O show lower levels compared to NW (*p*-value < 0.001), whereas UW subjects show lower levels compared to OW (*p*-value < 0.05). Total GM3 was higher in NW compared to OW (*p*-value < 0.01) and to O (*p*-value < 0.05) subjects. GM3 was higher in OW and O subjects compared to the UW (*p*-value < 0.05 in both comparisons) group.

A clear trend for decrement was found across different BMI percentiles in S1P levels, and statistically significant differences were observed comparing UW vs. O (*p*-value < 0.05) and NW vs. O (*p*-value < 0.05).

#### To Summarize:

- Cers: UW subjects were found to have lower levels of total Cers, cer (d18:1/22:0), and cer (d18:1/24:0) compared to NW. OW subjects showed lower levels of total Cers and all acyl Cers chains compared to NW and O. UW and OW subjects are characterized by lower levels for total Cers, Cer (d18:1/16:0), and Cer (d18:1/24:0).
- dhCers: Comparing UW and NW groups, lower levels of total SMs and SM (d18:1/22:0) were observed in NW. OW subjects showed lower levels of total dhCers when compared to NW and O groups.
- SMs: OW subjects showed higher levels of total SMs and all acyl SMs chains compared to their NW and O counterparts. OW subjects were also characterized by higher levels of SM (d18:1/22:0) compared to UW subjects only.
- HexCer, diHexCer, and GM3: NW subjects showed higher levels of HexCer, dihexCer, and GM3 compared to UW and O. UW subjects had lower levels of total dihexCer compared to OW subjects and lower levels of total GM3 compared both to OW and O groups. When comparing NW vs. OW, total HexCer and total GM3 were higher in NW subjects.
- S1P: a decrement in S1P levels was observed in O subjects compared to both UW and NW.

## 3. Discussion

This is the first study considering a sphingolipid pattern in relation to high-altitude hypoxia exposure. In previous reports, a discrepancy between the lipid and metabolic profile of Koya children, born and living at 3775 m a.s.l., compared with age and sex matched lowlanders [15,16], was described. This group was characterized by slightly high glucose compared to lowlanders, lower insulin level, lower HDL level, whereas TC/HDL-C, LDL-C/HDL-C, and triglycerides/HDL-C were higher in high-altitude children, and particularly in overweight and obese [8,15,16,17,37] children. An independent study performed in the San Pedro de Cajas district, located in the Central Andes of Peru at 4100 m a.s.l., showed similar results, with a high prevalence of hypertriglyceridemia (53.9%) and low HDL-C (45.3%) in indigenous descendants of the Amerindian populations, identifying these traits as characteristic of this population living at altitude [38]. In the present study, metabolic parameters show that mean levels of HDL-C are lower than the safety cut-off (45 mg/dL) for UW and O subjects, while triglycerides range from borderline (79–99 mg/dL) in UW, NW, and OW subjects to high (>110 mg/dL) in O subjects, confirming a particular dyslipidemic pattern in this group. More importantly, results from the present study identified the axis HDL-C, ceramide qualitative and quantitative variations, and S1P levels as related to hypoxia adaptation and body weight in Koya children, and indicated that total ceramide levels correlate directly with several biochemical parameters, whereas S1P correlate indirectly.

**Figure 5:**
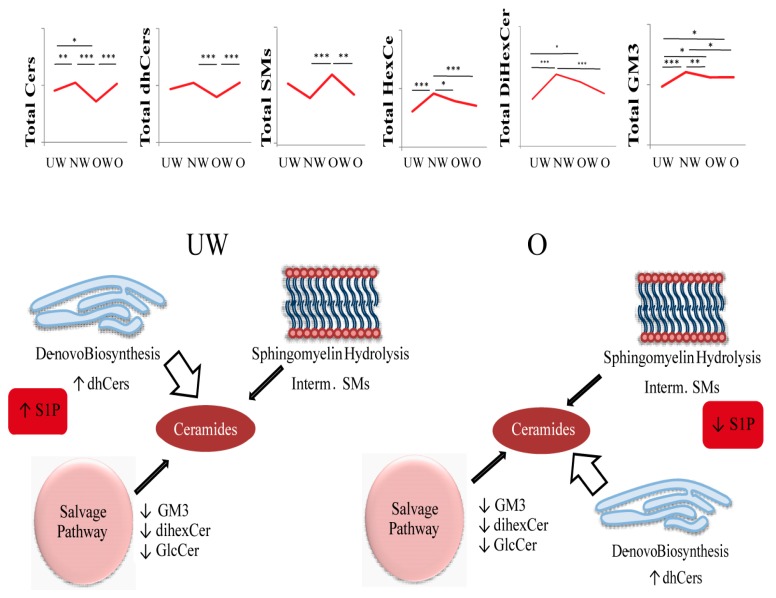
Summary of the altered ceramide-biosynthesis pathways. Different size arrows indicate the different involvement of Cers biosynthetic pathways in UW and O. LC-MS and LC-MS/MS results for total Cers, total dhCers, total SMs, total HexCer, total dihexCer, and total GM3. Data are expressed as pmol/mL in Log scale; *p* values are expressed as * *p*-value < 0.05, ** *p*-value < 0.01, and *** *p*-value < 0.001.

Figure 5 summarizes the levels of sphingolipids or glycosphingolipids in the four groups and indicates that UW and O subjects are characterized by similar glycosphingolipids levels, with S1P values varying, being high in UW subjects and low in O subjects.

In UW children, Cer levels seem to arise from *de novo* biosynthesis (being dhCers levels comparable to NW as S1P). Conversely, O subjects are characterized by similar levels of ceramides as NW, whereas S1P is significantly decreased, making this molecule a candidate as a central node in the metabolic adaptation to hypoxia of Koya children.

A previous important study demonstrated that increment of S1P is associated with increased capacity of oxygen delivery from erythrocytes, by increasing 2,3-diphosphoglycerate and activating enzymes involved in glycolysis [39]. In the circulation, 2/3 of the S1P is bound to HDL, carried out by the Apolipoprotein M (apoM), and 1/3 is bound to albumin [40,41]. S1P bound to apoM promotes vasorelaxation by phosphorylating endothelial nitric oxide synthase (eNOS) [42]. The eNOS is responsible for the generation of nitric oxide (NO) in the vascular endothelium and plays a crucial role in vascular tone [43] and homeostasis through NO. Further, eNOS uncoupling generates endothelial dysfunction due to superoxide anions and hydrogen peroxide overproduction. A previous study from our group demonstrated that the NO releasing donors causes ceramide accumulation in tumor cell lines, inducing a switching of the Akt hyperactivated signal leading to UPR (unfolded protein response) stress response [44]. There is a possibility that ceramide accumulation is also, in this case, associated with UPR activation. Further studies are required to elucidate the mechanism at the basis of ceramide levels of high-altitude living subjects.

Another study by our group on muscle tissues of Tibetans living at altitude and Tibetan lowlanders indicates an increment of e- and nitric oxide synthase (n-NOS) in muscle tissue and of 2,3-diphosphoglycerate (2,3-BPG). The latter is an erythrocyte-specific glycolytic intermediate, which facilitates O_2_ release found to be increased in animal models overexpressing S1P [39]. In animal models, intracellular S1P promoted increased production of 2,3-bisphosphoglycerate. In light of the present results, the authors can postulate that UW children utilizing S1P can activate the same biochemical pathway promoting oxygen delivery to maintain energy production and homeostasis, thereby keeping levels of ceramides as low as possible. Conversely, O children, characterized by lower levels of S1P, are unable to reduce ceramide levels; even if the salvage pathway is decreased, they may lose the capacity to adapt to a lack of oxygen, therefore activating the lipogenic and lipotoxic pathways [45,46,47,48].

Another point that characterizes UW and O children is the decreased levels of glycosphingolipids generated in the salvage pathway. The activation of Neuraminidase 3 (NEU3) sialidase in transgenic mice expressing NEU3 sialidase caused reduced tissue and circulating levels of GM3 [49]. This molecule was found increased in our studies on muscle tissue of animal models exposed to prolonged severe hypoxia [50]. In the present study, it can be speculated that decreased levels of GM3, Hexosylceramides, and dihexosylceramides can be associated with the activation of NEU 3 sialidase. The latter has been recently described as a target of HIF 2 alpha, and thus regulated by hypoxia and associated with obesity [51].

Concerning levels of different ceramide acyl-chains, it can be hypothesized that the elevated C16:0-ceramide levels observed in O subjects can contribute to insulin resistance [23,52]. Ceramides substantially upregulate fatty acid uptake and synthesis through direct or indirect mechanisms and could be central modulators of obesity and hepatic steatosis [53]. Furthermore, specific ceramide synthases (in this case, CerS6) have been associated with weight gain and glucose intolerance [23]. Several studies addressed the issue of the role of S1P in inflammation. In this context, O are characterized by low levels of S1P compared to UW. It has been described that S1P improves energy homeostasis in obesity, particularly in the early phase, acting as protective molecule to control overweight and inflammation [54]. This observation is in agreement with our results, since in OW levels of S1P are high compared to O. Furthermore, this molecule, in conjunction with its receptor, decreases endothelial inflammation and promotes cardioprotection mediated by HDL-Apo-M-S1P binding and release [55,56,57]. Further studies in this direction are required.

The major limitations of this study are the restricted number of subjects and the absence of a verification study, which will require an independent group of subjects. Further data based on 2,3-BPG, pH, partial pressure of carbon dioxide (PCO_2_), of oxygen (PO_2_), and haemoglobin-O_2_ saturation (SO_2_) will be necessary to determine the abnormal O_2_ affinity or altered metabolism of RBC [58]. This data, in conjunction with lipemia, could be supportive of our results. Another important aspect that has not been addressed in this study for unavailability of fresh serum samples is the study of RNAs from peripheral blood mononuclear cells (PBMCs) for the quantitative assessment of transcripts of enzymes involved in ceramide pathways and of circulating cytokines or adipokines. Studies in this direction are ongoing and are aimed at opening new avenues in the complex field of hypoxia tolerance in high-altitude dwellers.

In conclusion, ceramides levels and S1P are the primary targets of hypoxia adaptation in populations born and living at high altitude. An important point highlighted in this study is that S1P can be a circulating biomarker of hypoxia tolerance and a valuable marker of increased risk of metabolic syndrome and cardiac dysfunction, being directly quantifiable in blood by selected reaction monitoring (SRM) mass spectrometry.

## 4. Materials and Methods

### 4.1. Participants and Sample Collection

Fifty-nine children (age 5–13 years) from San Antonio de Los Cobres (3775 m a.s.l.), who were previously enrolled [15], were classified as UW (<5th percentile), NW (5th to < 85th percentile), OW (85th to < 95th percentile), or O (≥95th percentile) on the basis of their body mass percentiles, accordingly to US Centers for Disease Control and Prevention (CDC) norms [59]. Children’s anthropometry—age, gender, weight (kg) and height (cm)—are indicated in Table 1. TC (mg/dL), HDL-C (mg/dL), LDL-C (mg/dL), Vit. D, ng/mL), fasting glucose (Glycaemia, mg/dL), fasting insulin (Insulin, µU/mL), and triglycerides TG, mg/dL) were assessed, while BMI, TC/HDL-C ratio, and Homeostatic model assessment of insulin resistance were calculated as follows: BMI was calculated as weight (kg) divided by height squared (m); TC/HDL-C ratio was calculated as TC(mg/dL)/HDL-C(mg/dL); while HOMA-IR was calculated as Glycaemia (mg/dL) × Insulin/405. Blood samples were collected after 10-h fasting. Fasting glucose was measured by glucose oxidase, while serum lipids were assessed with Architect c 16,000 instrument (Toshiba, Kanagawa, Japan). Chemiluminiscent was utilized for the detection of fasting insulin levels and serum Vitamin D (Abbott Laboratories, Chicago, IL, USA).

The study was carried out following the rules of the Declaration of Helsinki and was approved by the Human Rights Committee of the Salta Health Ministry (project number: 4518; date of approval: 8 November 2010). Each caregiver and child gave written informed consent after an explanation of the study and before its initiation.

### 4.2. Reagents and Chemicals

Methanol, LC-MS grade water, 3,5-Di-tert-4-butylhydroxytoluene (BHT), and ammonium formate were from Sigma-Aldrich (Saint Louis, MO, USA). Ethanol and HPLC analytical grade chloroform were, respectively, from J.T. Baker (Center Valley, PA, USA) and Carlo Erba (Cornaredo, MI, Italy). Potassium hydroxide was from Merk Millipore (Burlington, MA, USA). Acetic and formic acid were from Fluka-Analitical (Honeywell, Morris Plains, NJ, USA).

Sphinganine (d17:0), sphinganine-1-phosphate (d17:0), C12 Ceramide, Sphingomyelin (d18:1/12:0), and Glucosyl (β) C12 Ceramide were from were from Avanti Polar Lipids (Alabaster, AL, USA).

### 4.3. Lipid Extraction

Sphingolipids were extracted from sera according to a previous study [60], with minor modification. Briefly, 0.1 mL of serum was mixed with 0.1 mL of ultrapure water and 1.5 mL of Methanol/chloroform 2:1, and fortified with internal standards 200 pmol: Sphinganine (d17:0), sphinganine-1-phosphate (d17:0), C12 Ceramide (d18:1/12:0), C12 Sphingomyelin (d18:1/12:0), and Glucosyl (β) C12 Ceramide. Samples were briefly sonicated and heated at 48 °C overnight. Then, 0.15 mL of KOH 1 M in methanol was added to every sample, and after 2-h incubation at 37 °C, the solution was neutralized with 0.15 mL of acetic acid 1 M and dried with Speedvac. Samples were then resuspended in 0.5 mL of methanol and transferred to a clean Eppendorf tube. Samples were dried, resuspended in 0.15 mL of methanol, and centrifuged for 3 min at 10,000× *g*. Liquid phases were collected in UPLC glass vials and stored at −80 °C.

### 4.4. UPLC-MS for Sphingolipids

Liquid chromatography-mass spectrometer configuration included a Waters Acquity UPLC system linked to a Waters LCT Premier Orthogonal Accelerated Time of Flight Mass Spectrometer (Waters, Millford, MA, USA). The instrument operated in positive or negative electrospray ionization mode. Full scans were obtained in a window, ranging from 50 to 1500 Da. Accuracy and reproducibility were maintained employing an independent reference spray via LockSpray. First, 10 µL of sphingolipid extract were injected and separated on an analytical column, kept at 30 °C, 100 mm × 2.1 mm id, 1.7 µm C8 Acquity UPLC BEH (Waters), using the following linear gradient: 0.0 min: 80% B; 3 min: 90% B; 6 min: 90% B; 15 min: 99% B; 18 min: 99% B; 20 min: 80% B, at 0.3 mL/min flow rate. Phase B consisted of 1 mM ammonium formate in methanol, 0.05 mM formic acid, while phase A was 2 mM ammonium formate in H_2_O, with 0.05 mM formic acid. Sphingolipids quantification was carried out using the ion chromatogram obtained for each compound using 50 mDa windows. The linear dynamic range was determined by injection of standard mixtures. Positive identification of compounds was based on the accurate mass measurement, with an error <5 ppm and its retention time, compared to that of a standard (± 2%). Mass spectra were analyzed by MassLynx™ 4.1 Software (Waters, Millford, MA, USA), and lipids were annotated as lipid subclasses as follows (sphingosine backbone/number of carbon atoms of the fatty acid: number of unsaturations of the fatty acid).

### 4.5. MRM Analysis for S1P

S1P was quantified using a Xevo TQ-MS mass spectrometer (Waters, Millford, MA, USA), where 10 µl of sphingolipid extract were injected and separated on an analytical column, kept at 30 °C, 100 mm × 2.1 mm id, 1.7 µm C8 Acquity UPLC BEH (Waters), with the following gradient: 0.0 min: 80% B; 3 min: 90% B; 6 min: 90% B; 9 min: 99% B; 12 min: 99% B; 14 min: 80% B, at 0.3 mL/min flow rate. Phase A and phase B were the same as above. An electrospray interface operating in positive ion mode was employed to obtain MS/MS spectra by acquiring MRM transitions of: C17 sphinganine-1-phosphate, 368.4–252.4, collision energy 18 eV; and S1P, 380.4–264.4 Da, collision energy 16 eV. Mass spectra were analyzed by MassLynx™ 4.1 Software.

### 4.6. Statistical Analysis

Participants were grouped according to their BMI percentiles and their characteristics were described using median and interquartile range (if continuous) or counts and percentages (if categorical). Comparison of age, weight, height, BMI, TC, HDL-C, CT/HDL, LDL-C, Vitamin D, glycaemia, HOMA-IR, insulin, and TG were investigated between groups using One way ANOVA with Bonferroni’s correction if data were normally distributed, otherwise the Kruskal-Wallis’ with Dunn’s correction was adopted. Correlation tables for sphingolipids and biochemical parameters were obtained using Pearson’s or Spearman’s correlation, when data were parametrically or not distributed, respectively. Differences in Cers, dhCers, SMs, dhSMs, HexCers, diHexCers, GM3, and S1P from LC-MS and LC-MS/MS quantitative data were assessed among groups.

Statistical analyses were performed using SigmaPlot software version 12.0 and Prism software version 7.0.

## Figures and Tables

**Table 1:** Study participant’s anthropometry and biochemical assessment. Characteristics are described using median and interquartile range (if continuous) or counts and percentages (if categorical). One way ANOVA with Bonferroni’s correction was employed if data were normally distributed, otherwise Kruskal-Wallis’ with Dunn’s correction was adopted. (* represent *p*-value for UW, **^+^** represent *p*-value for NW, **^y^** represent *p*-value for O) * *p*-value < 0.05, ** *p*-value < 0.01, *** *p*-value < 0.001, **^+^** *p*-value < 0.05, **^++^** *p*-value< 0.01, **^+++^** *p*-value< 0.001, **^yy^** *p*-value < 0.01.

Anthropometric and Biochemical Parameters	UW	NW	OW	O
N.	7 (43.7 %)	30 (44.1 %)	13 (65 %)	9 (50%)
Age	11 (8.5/11)	9 (8/11)	11 (10/11)	9 (8/9)
Weight (Kg)	25.2 (21.8/27.1)	27.2 (22.6/32.6)	45 (37.3/49.5) ***^,+++^	38.6 (37.6/42) **^,++^
Height (Cm)	134 (124/138.5)	129.5 (121.7/140.7)	145 (134/149)	130 (124/137)
BMI	14 (13.7/14.3)	15.765 (14.9/16.9)	20.9 (18.6/22) ***^,++^	23.9 (22.5/25.6) ***^,+++^
Gender (M)	4 (57,1 %)	15 (50 %)	7 (53,8 %)	4 (44,4 %)
TC (mg/dL)	148 (128.5/163)	170 (166/180.5)	159 (140/170)	174 (148/193)
HDL-C (mg/dL)	43 (41/53)	50.5 (45/59.5) ^yy^	45 (40/50)	42 (32/46)
TC/HDL-C	3.1 (2.8/3.3)	3.35 (2.9/3.7)	3.7 (3.2/3.9)	4.1 (3.7/4.8) *^,++^
LDL-C (mg/dL)	81 (74/90.5)	99.5 (94.2/104.7)	85 (81/100)	101 (89/104)
Vit. D (ng/mL)	14.2 (14/16.1)	17.2 (14.6/21.2)	16.2 (12.7/18.8)	15.8 (14.3/18.3)
Glycaemia (mg/dL)	88 (82/92)	83.5 (79.5/87.7)	85 (81/88)	84 (81/88)
HOMA-IR	1 (0.9/1.2)	0.9 (0.6/1.1)	1.7 (1.1/1.7) ^+^	1.5 (1.3/2) ^++^
Insulin (µU/mL)	5.1 (3.9/5.6)	4.4 (3/5.6)	7.7 (5.5/8.7) ^+^	7.2 (6.4/9.3) ^++^
TG (mg/dL)	83 (72.5/95.5)	97 (78.2/113.7)	90 (86/122)	129 (97/143)

## Supplementary Materials

Supplementary materials can be found at https://www.mdpi.com/1422-0067/20/11/2835/s1.

## Abbreviations

UW	Underweight
NW	Normal weight
OW	Overweight
O	Obese
LC-MS/MS	Liquid Chromatography Mass Spectrometry/Mass spectrometry
Cers	Ceramides
S1P	Sphingosine 1 phosphate
SMs	Sphingomyelins
HDL-C	High density lipoprotein-cholesterol
LDL-C	Low density lipoprotein-cholesterol
A.s.l	Above sea level
HIF	Hypoxia inducible factor
EPAS1	Endothelial PAS Domain Protein 1
EGLN1	Egl-9 Family Hypoxia Inducible Factor 1
NOS2A	Nitric oxide synthase 2
CH	Chicoana
SAC	San Antonio de los Cobres
BA	Buenos Aires
CerS	Ceramide Synthases
SphK	Sphingosine Kinase
ER	Endoplasmic reticulum
HOMA-IR	Homeostatic model assessment of insulin resistance
TC	Total Cholesterol
TG	Triglycerides
Vit. D	Vitamin D
dhCers	Dihydroceramides
HexCer	Hexosylceramides
DiHexCer	DiHexosylceramides
GM3	Monosialodihexosylganglioside
BMI	Body Mass Index
apoM	Apolipoprotein M
eNOS	Endothelial nitric oxide synthase
NO	Nitric oxide
UPR	Unfolded protein response
2,3-BPG	2,3-diphosphoglycerate
NEU3	Neuraminidase 3
PCO_2_	Partial pressure of carbon dioxide
PO_2_	Partial pressure of oxygen
SO_2_	Haemoglobin-O2 saturation
n-NOS	Nitric oxide synthase
PBMCs	Peripheral blood mononuclear cells
SRM	Selected Reaction Monitoring
CDC	Centers for Disease Control and Prevention
BHT	3,5-Di-tert-4-butylhydroxytoluene
