# Supplementary material for: Regulation of Serum Sphingolipids in Andean Children Born and Living at High Altitude (3775 m)
Source: Int J Mol Sci. 2019 Jun 11;20(11):2835. doi: 10.3390/ijms20112835 (PMC6600227; doi:10.3390/ijms20112835)
Supplement: Supplementary file 1 [file ijms-20-02835-s001.zip › Supplementary Table 1.pdf]

	Total Cholesterol		HDL-C		CT/HDL		LDL-C		Vit. D		Glycemia		HOMA-IR		Insulin		TG	
	r	p	r	p	r	p	r	p	r	p	r	p	r	p	r	p	r	p
UW Total Cers	-0.303	0.509	0.066	0.888	-0.373	0.411	-0.440	0.323	-0.292	0.526	-0.393	0.383	-0.320	0.485	-0.310	0.498	-0.026	0.957
NW Total Cers	0.091	0.632	-0.238	0.206	0.319	0.085	0.107	0.574	-0.400	0.028	0.035	0.852	0.389	0.036	0.418	0.021	0.379	0.039
OW Total Cers	0.721	0.005	**0.335**	0.263	0.280	0.355	0.619	0.024	-0.312	0.299	0.028	0.928	-0.097	0.753	-0.109	0.723	0.359	0.228
O Total Cers	0.440	0.237	-0.321	0.399	**0.544**	0.135	-0.268	0.486	-0.035	0.929	0.683	0.043	0.477	0.195	0.420	0.260	0.3285	0.4269
UW Total SMs	0.478	0.278	0.854	0.015	-0.368	0.417	0.311	0.497	-0.052	0.912	0.167	0.721	-0.267	0.564	-0.281	0.541	-0.176	0.706
NW Total SMs	-0.093	0.624	-0.049	0.798	0.024	0.901	-0.029	0.880	-0.091	0.634	-0.095	0.616	-0.257	0.171	-0.240	0.202	-0.040	0.834
OW Total SMs	0.721	0.005	0.603	0.029	0.027	0.931	0.655	0.015	-0.064	0.834	0.055	0.859	0.189	0.537	0.188	0.538	-0.010	0.975
O Total SMs	0.225	0.560	0.264	0.492	**-0.259**	0.498	0.183	0.637	0.425	0.254	0.152	0.696	-0.152	0.696	-0.203	0.601	-0,018	0,975
UW Total S1P	0.384	0.396	0.400	0.374	0.006	0.990	0.385	0.394	0.557	0.194	-0.386	0.392	-0.512	0.241	-0.459	0.301	-0.166	0.723
NW Total S1P	-0.183	0.333	-0.375	0.041	0.350	0.058	0.052	0.786	0.015	0.936	-0.010	0.957	0.049	0.798	0.014	0.941	0.205	0.278
OW Total S1P	**-0.528**	0.067	-0.176	0.566	-0.376	0.205	-0.691	0.011	0.077	0.806	0.271	0.368	0.187	0.539	0.132	0.669	0.344	0.249
O Total S1P	-0.348	0.358	-0.491	0.180	**-0.008**	0.988	-0.464	0.208	0.637	0.065	0.410	0.273	0.458	0.215	0.451	0.224	**0.360**	0.339

Supplementary Table 2. p-values from two tailed test, r value from Pearson's correlation (regular) or from Spearman's correlation (bold).
