# Supplementary figures and images for: Regulation of Serum Sphingolipids in Andean Children Born and Living at High Altitude (3775 m)
Source: Int J Mol Sci. 2019 Jun 11;20(11):2835. doi: 10.3390/ijms20112835 (PMC6600227; doi:10.3390/ijms20112835)

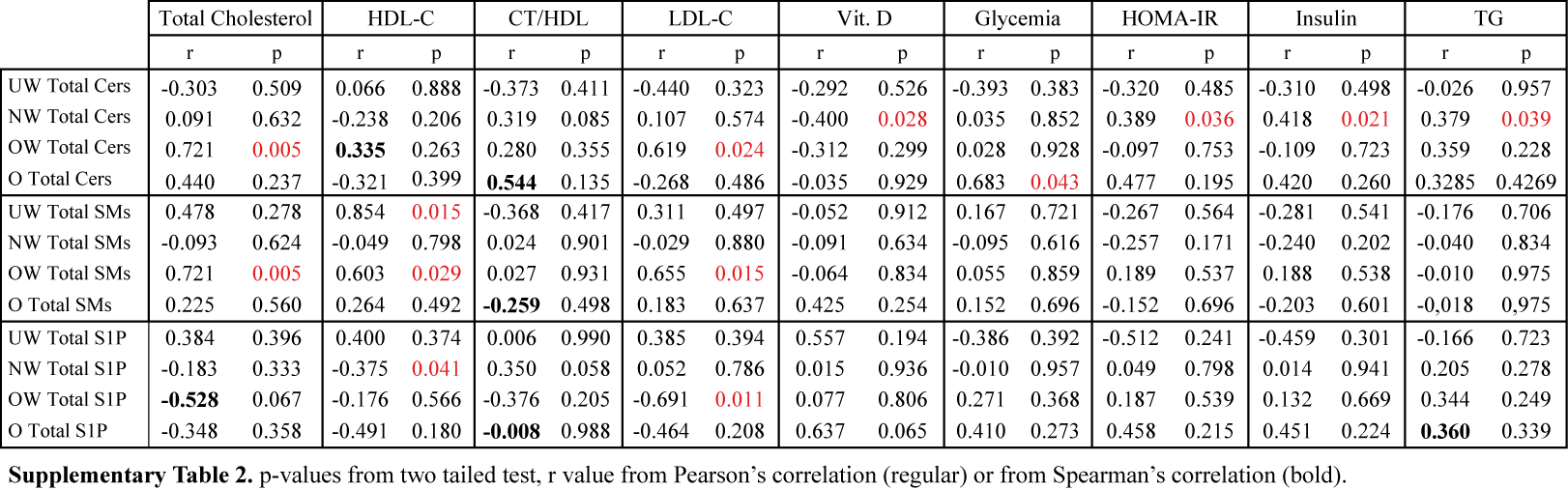

Supplement: Supplementary file 1 [file ijms-20-02835-s001.zip › Supplementary Table 1.png]
